# Estudio clínico de seguridad, aceptabilidad galénica y eficacia del preparado GV-328 en la aftosis oral infantil

**DOI:** 10.1016/j.aprim.2022.102311

**Published:** 2022-02-25

**Authors:** Francisco Guinot Jimeno, Jessica Nicole Fernández Sencion, Luis Giner Tarrida, Ana Veloso Durán

**Affiliations:** Departamento de Odontopediatría, Facultad de Odontología, Universitat Internacional de Catalunya, Barcelona, España

Con el objetivo de evaluar la seguridad, aceptabilidad galénica y eficacia del preparado GV-328, actualmente comercializado como complemento dietético, para el tratamiento de la lesión aftosa en los niños, se diseñó un estudio abierto prospectivo, aprobado por el Comité Ético de Investigación Clínica (ODP-ECL-2017-01) de la Universitat International de Catalunya (UIC) y registrado en ClinicalTrials.gov (Identificador: NCT04677062). Todos los padres o tutores de los niños que participaron en el estudio dieron su consentimiento informado de manera voluntaria y escrita. El estudio cumple con los principios éticos en investigación médica en seres humanos establecidos en la Declaración de Helsinki (última actualización octubre 2013).

Se incluyeron niños de entre 3 y 13 años atendidos en el Servicio de Odontopediatría de la Clínica Universitaria de Odontología de la UIC afectados por aftas bucales con un máximo de 48 h de evolución (excluyendo las lesiones múltiples o traumáticas y pacientes que estuvieran tomando medicación concomitante y los pacientes diabéticos)^1^. Todos fueron tratados con el preparado GV-328, en pastillas con ácido hialurónico y cinc, y un soporte de plástico para evitar que se trague de golpe^2^. Se disuelve lentamente con la saliva como si fuera un caramelo bucal, y actúa tópicamente en la zona afectada. Tanto el contenido en ácido hialurónico (5 mg/pastilla) como en cinc (1 mg/pastilla) pueden considerarse muy seguros para su uso en la población infantil^3^, ^4^. El tratamiento en estudio (entre 4 y 6 pastillas al día según la edad) se realizó durante 4 días.

Se registraron mediante escalas tipo Likert las valoraciones sobre la intensidad percibida de dolor, limitación funcional, y edema de la mucosa marginal, así como del tamaño de la lesión (< 0,5, 0,5-1,0 o > 1,0 cm)^5^, ^6^. Se realizó una fotografía en las visitas inicial y final, y se recogieron los juicios globales de los investigadores y padres, sobre la eficacia, tolerabilidad, aceptabilidad, facilidad de aplicación y sabor (según una escala de bueno, regular o malo).

Se incluyeron 33 pacientes (11 niños [33%] y 22 niñas [67%]). La edad media fue de 7,8 (DE: 1,4) años, que utilizaron de media 5 pastillas al día (4,8 [DE: 0,7]).

El 100% de los casos mostró mejoría en la visita final, con reducciones estadísticamente significativas (p < 0,05) en la intensidad del dolor (desde el primer día y a diario), la limitación funcional y el edema tras la aplicación del preparado GV-328 (tabla 1).

**Tabla 1:** Evolución del dolor, la limitación funcional y el edema durante el tratamiento con GV-328

	Media	DE	Mediana	Q1-Q3	Valor de p
**Valoración de los investigadores**^a^					
*Dolor*					
Inicial (día 1)	4,3	0,6	4	4-5	< 0,01
Final (día 5)	0,7	1	0	0-1	

*Limitación funcional*					
Inicial (día 1)	3,1	0,9	3	2-4	< 0,01
Final (día 5)	0,4	0,8	0	0-1	

*Edema*					
Inicial (día 1)	2,6	1,1	2	2-4	< 0,01
Final (día 5)	0,6	0,6	1	0-1	

**Valoración de los padres/pacientes**^b^					
*Dolor*					
Día 1 - Antes	4,3	0,8	4	4-5	< 0,01
Día 1 - Después	3,6	0,8	4	3-4	
Día 2 - Antes	3,2	0,9	4	2-4	< 0,01
Día 2 - Después	2,6	1,3	3	2-3	
Día 3 - Antes	2,4	0,9	3	2-3	< 0,013
Día 3 - Después	1,9	1,2	2	1-3	
Día 4 - Antes	1,4	1,2	2	0-2	< 0,01
Día 4 - Después	1,0	1,1	0	0-2	

Las columnas resumen la media, desviación estándar (DE), mediana, rango cuartiles 1 y 3 (Q1-Q3), y el valor de p (Wilcoxon datos pareados). Total 33 casos.

a Escala de intensidad de los síntomas de 0 «ausente» a 5 «máxima», en las visitas inicial y final.

b Escala de caras de Wong-Baker (intensidad de 0 «ausente» a 5 «máxima»), antes de la primera pastilla y después de la última del día, durante los 4 días de tratamiento.

El tamaño de las aftas también se redujo de forma estadísticamente significativa (p < 0,01) respecto a la visita inicial. El 39% de los casos (n = 13) alcanzó la cicatrización completa y el 61% restante (n = 20), presentó al finalizar un diámetro de la lesión menor de 0,5 cm.

El perfil de seguridad fue bueno. No se reportaron efectos adversos graves. Tres casos (9%) refirieron náuseas al inicio, relacionadas con el sabor del producto.

Se realizó un análisis comparativo de los juicios globales de investigadores y de padres en cuanto a la eficacia, tolerabilidad, aceptabilidad, facilidad de aplicación y sabor del preparado, sin encontrar diferencias estadísticamente significativas entre ambos en ninguna de las variables evaluadas. En todos los casos, las valoraciones fueron excelentes.

De los resultados del estudio se deduce que el preparado GV-328, presentó una alta eficacia en el alivio del dolor, en la reducción del tiempo de cicatrización y en la mejora de la limitación funcional desde el primer día de tratamiento, con un buen perfil de seguridad.

Tanto los investigadores como los padres de los pacientes consideraron en su mayoría como buena, la eficacia, la tolerabilidad, la aceptabilidad y la facilidad de aplicación.

La presentación del producto en forma de pastilla-caramelo para desleír en la boca, facilitó la aplicación tópica del tratamiento en la población pediátrica.

Una limitación del estudio es la ausencia de grupo de control en el mismo. Aunque el tiempo de curación de nuestro estudio fue inferior en general al de remisión espontánea (1 o 2 semanas), para poder afirmar la reducción de la duración del proceso aftoso, sería necesario realizar un estudio que incluyera un grupo control que siguiera el proceso natural o bien un grupo placebo.

## Financiación

El trabajo ha sido financiado por Laboratorios Viñas.

## Conflicto de intereses

Los autores declaran no haber recibido honorarios por la realización del trabajo.
